# Estimating Angle-of-Arrival and Time-of-Flight for Multipath Components Using WiFi Channel State Information

**DOI:** 10.3390/s18061753

**Published:** 2018-05-29

**Authors:** Afaz Uddin Ahmed, Reza Arablouei, Frank de Hoog, Branislav Kusy, Raja Jurdak, Neil Bergmann

**Affiliations:** 1 School of Information Technology and Electrical Engineering, University of Queensland, St Lucia, QLD 4072, Australia; afaz.ahmed@uqconnect.edu.au (A.U.A.); bergmann@itee.uq.edu.au (N.B.); 2 Data61, Commonwealth Scientific and Industrial Research Organisation (CSIRO), Pullenvale, QLD 4069, Australia; reza.arablouei@data61.csiro.au (R.A.); frank.dehoog@data61.csiro.au (F.d.H.); brano.kusy@data61.csiro.au (B.K.); raja.jurdak@data61.csiro.au (R.J.)

**Keywords:** angle of arrival estimation, indoor localization, joint AoA–ToF estimation, matrix pencil, multipath propagation, MUSIC algorithm, time of flight estimation, WiFi channel state information

## Abstract

Channel state information (CSI) collected during WiFi packet transmissions can be used for localization of commodity WiFi devices in indoor environments with multipath propagation. To this end, the angle of arrival (AoA) and time of flight (ToF) for all dominant multipath components need to be estimated. A two-dimensional (2D) version of the multiple signal classification (MUSIC) algorithm has been shown to solve this problem using 2D grid search, which is computationally expensive and is therefore not suited for real-time localisation. In this paper, we propose using a modified matrix pencil (MMP) algorithm instead. Specifically, we show that the AoA and ToF estimates can be found independently of each other using the one-dimensional (1D) MMP algorithm and the results can be accurately paired to obtain the AoA–ToF pairs for all multipath components. Thus, the 2D estimation problem reduces to running 1D estimation multiple times, substantially reducing the computational complexity. We identify and resolve the problem of degenerate performance when two or more multipath components have the same AoA. In addition, we propose a packet aggregation model that uses the CSI data from multiple packets to improve the performance under noisy conditions. Simulation results show that our algorithm achieves two orders of magnitude reduction in the computational time over the 2D MUSIC algorithm while achieving similar accuracy. High accuracy and low computation complexity of our approach make it suitable for applications that require location estimation to run on resource-constrained embedded devices in real time.

## 1. Introduction

Modern radio communication devices are not just transceivers. They are also sensors of the characteristics of the propagation environment within which they operate. The information collected about the radio propagation properties of the environment is often referred to as the channel state information (CSI). The main use for the sensed CSI is to adaptively optimize the communication throughput according to the environment. However, the CSI can also be utilized to estimate the geolocation (position) of the end-user communication devices, such as mobile phones, hence the position of the individuals using the devices. This is of particular importance as the position information plays an essential role in most context-aware services and systems [1,2].

WiFi CSI-based indoor localization using commercial network interface card (NIC) devices has recently received substantial attention [3]. Common WiFi standards, including 802.11a, 802.11n, and 802.11ac, rely on the orthogonal frequency division multiplexing (OFDM) modulation technique that transmits data simultaneously on multiple frequencies known as subcarriers. The CSI data collected on all of the subcarriers provides valuable information that can be utilized to localize the source. However, in a typical indoor environment, the received signal is the superposition of four to eight dominant multipath components traveling along different paths before reaching the access point [4]. Therefore, an effective indoor localization technique has to appropriately resolve the multipath components and identify the direct path. Previous studies have shown that the receiver must include antenna arrays with more antennas than the number of multipath components to be able to directly resolve the angle of arrival (AoA) and time of flight (ToF) of all multipath components on a single frequency [5]. This creates a fundamental constraint for estimating the AoA and ToF of multipath components using commercial WiFi devices.

The advantage of OFDM schemes is that they allow the CSI data to be measured on multiple frequencies (subcarriers). Therefore, they help overcome the limitation of the single-frequency antenna arrays [6]. Specifically, subspace-based parameter estimation methods can be used to jointly estimate the AoA and ToF of the multipath components from the CSI data. The multiple signal classification (MUSIC) algorithm can be used to solve such joint AoA and ToF estimation problems [7,8,9]. However, the MUSIC algorithm performs a two-dimensional (2D) grid search, which is often computationally expensive.

In this paper, we propose estimating the AoA and ToF of the multipath components from the WiFi CSI data using a search-free subspace-based algorithm, inspired by the matrix pencil technique. This technique was first proposed in [10] to estimate the angle of arrival of a signal at a rectangular antenna array in 3D space. Conceptually, this is a similar problem, where two parameters (namely azimuth and incidence angles) are estimated in a 2D subspace. It is shown in [10] that the problem can be solved in two steps using 1D estimation techniques. In [11], this work was further improved through the modified matrix pencil (MMP) algorithm that ensures the 1D values are matched in a correct order.

We consider a system with three antennas and a commodity WiFi chip that can measure CSI over multiple OFDM subcarriers for all three antennas. We apply the MMP algorithm to the CSI data of all antennas and subcarriers to estimate the AoA and ToF parameters associated with the dominant multipath components. In the process, we arrange the CSI values to form an enhanced CSI data matrix. This matrix facilitates the use of the matrix pencil concept to identify the signal subspace matrix. The signal subspace matrix is converted to a pair of matrices to estimate the eigenvalues that represent the parameter estimates in one dimension. A permutation is subsequently applied to the signal subspace matrix to obtain the parameter estimates in the second dimension. We show that the MMP algorithm reduces the solution time compared to the 2D MUSIC algorithm by nearly two orders of magnitude while delivering similar estimation accuracy. Furthermore, we identify an incorrect behavior of the MMP algorithm in the case where two multipath components have the same AoA. We show that the problem can be addressed by estimating the ToF first in the MMP algorithm. Finally, we propose a multi-packet CSI aggregation method that uses the CSI values from several packets to improve both accuracy and speed of the joint AoA and ToF estimation for multipath components.

We evaluate the performance of the MMP algorithm via numerical simulations using the 2D-MUSIC algorithm as a performance benchmark. In terms of time complexity, MMP performs around 200 times faster than 2D-MUSIC with the same estimation accuracy. We assess the performance of MMP in different scenarios with and without noise and provide a concise summary. We consider the case when the order of AoA and ToF estimation is altered. In addition, we show that the proposed packet mutli-packet CSI aggregation method considers CSI from multiple packets to increase the signal-to-noise ratio (SNR) and then perform only one estimation step making the process faster and more accurate.

The main contributions of this work can be summarized as:

- proposal of a fast algorithm that estimates the AoA and ToF of the dominant multipath components from WiFi CSI data based on the MMP algorithm;
- demonstrating the advantage of changing the estimation order in the MMP algorithm;
- introducing a multi-packet CSI aggregation method that utilizes the information provided by multiple packets to deliver better estimation performance;
- numerical analysis in different scenarios illustrating the performance of the AoA and ToF estimation using the MMP and 2D MUSIC algorithms, effects of change of the estimation order in MMP, and benefits of the mutli-packet CSI aggregation method.

## 2. Related Work

Joint AoA and ToF estimation techniques have been developed in previous studies targeting applications in radar, sonar, and wireless communication [12,13,14,15,16,17,18,19,20,21,22]. Some of these techniques utilize the channel response to detect a higher number of multipath signals [6,18,22,23]. The initial works on joint AoA–ToF estimation for multipath components focused on high-resolution subspace-based algorithms operating on rectangular [10,11,24,25,26,27] and other 2D antenna array structures [13,14,18,28]. They use subspace-based algorithms such as 2D MUSIC [16,18,21,26,27,29], 2D ESPRIT (estimation of signal parameters via rotational invariance) [13,14,18,22,23,24,27,28], and 2D matrix pencil [10,11,25]. The 2D MUSIC performs three rounds of search. First, for a fixed ToF, it searches in a range of AoAs. Second, the ToF is varied in a given range. Finally, estimation is performed finding the peaks of the MUSIC spectrum. A recent indoor localization algorithm, called SPOTFI [6], uses the 2D MUSIC to estimate the AoA and ToF of the multipath components from the CSI readings. However, for better performance in noisy indoor environments, it considers the CSI readings from multiple packets. There is an inherent trade-off between the computational complexity and estimation accuracy of the 2D MUSIC algorithm governed by its grid resolution [30]. Moreover, it needs to find the peaks of the MUSIC spectrum on the grid, which is generally a slow process. There have been several proposals to accelerate the peak-search process of the 2D MUSIC algorithm. In [21], the author proposed three steps for searching. The technique proposed in [28], performs a two-step peak search. The first search is within a low-resolution grid followed by a second search on a grid with higher resolution around the found peaks. A similar approach is presented in [29]. In [31], a quadratic optimization is proposed using the least-squares method. However, none of the mentioned techniques is completely free of a classic search mechanism.

Unlike 2D MUSIC, the 2D matrix pencil algorithm is search-free and uses two 1D estimation processes to solve the associated 2D problem. The matrix enhanced matrix pencil (MEMP) algorithm [10] is a matrix-pencil-based technique that solves the 2D angle estimation problem over a rectangular antenna array. This algorithm creates an enhanced matrix using the signals received at the rectangular array where the number of array elements is higher than the number of multipath components (or signal sources). The modified matrix pencil (MMP) algorithm [11] improves the MEMP algorithm by including a pairing method that gives the solution of the 2D problem in the correct order.

The MMP algorithm uses the signal matrix received at a rectangular array to construct an enhanced matrix and create the required matrix pencil. After finding the value of one parameter, it shuffles the order of the signal subspace to estimate the other parameter. Performing two 1D estimation processes without any grid search makes this algorithm significantly faster than the 2D MUSIC algorithm. However, the MMP algorithm is developed for the setup where the number of antennas in the array is higher than the number of multipath components. In this paper, we use the MMP algorithm for estimating the AoA and ToF of multipath components using the WiFi CSI data, a task that is at the core of many indoor localization techniques such as SPOTFI, which uses the 2D MUSIC algorithm for this purpose. Our work replaces one of the two spatial dimensions in a rectangular antenna array with the CSI readings from multiple subcarriers, hence enabling the combination of 1D MMP solutions for CSI-based indoor localization with fewer antennas than the anticipated multipath components. Note that the 2D root-MUSIC and 2D ESPRIT algorithms are also in general more computationally efficient than the 2D MUSIC algorithm as they do not need any grid search. Root-MUSIC requires finding the roots of a polynomial equation that is expressed using the eigenvectors of the array data correlation matrix that correspond to the noise subspace [32]. ESPRIT performs estimation by determining a rotation operator using the eigenvectors of the correlation matrix corresponding to the signal subspace. However, the requirement of computing the correlation matrix makes root-MUSIC and ESPRIT computationally more demanding than the matrix-pencil-based methods, which work directly with the data [33,34].

## 3. MMP for Joint AoA–ToF Estimation

In this section, we present our MMP-based approach for estimating the AoA and ToF of mutlipath components. We first present the system model and our solution, then discuss how to pair the ToF and AoA estimates followed by a discussion of the challenges pertaining to the order of the AoA and ToF estimation.

### 3.1. System Model

We assume a 2D environment for the AoA–ToF estimation. In indoor localization, the elevation is not important and the receiver and transmitter are considered to be on the same plane. In [10,11], the data matrix is constructed using signals from a rectangular antenna array. In our approach, we adopted a similar strategy in terms of exploiting the structure inherent to the CSI data matrix. We construct an enhanced matrix using the CSI data so that the matrix pencil can be applied efficiently to estimate the 2D parameters of AoA and ToF.

**Figure 1:**
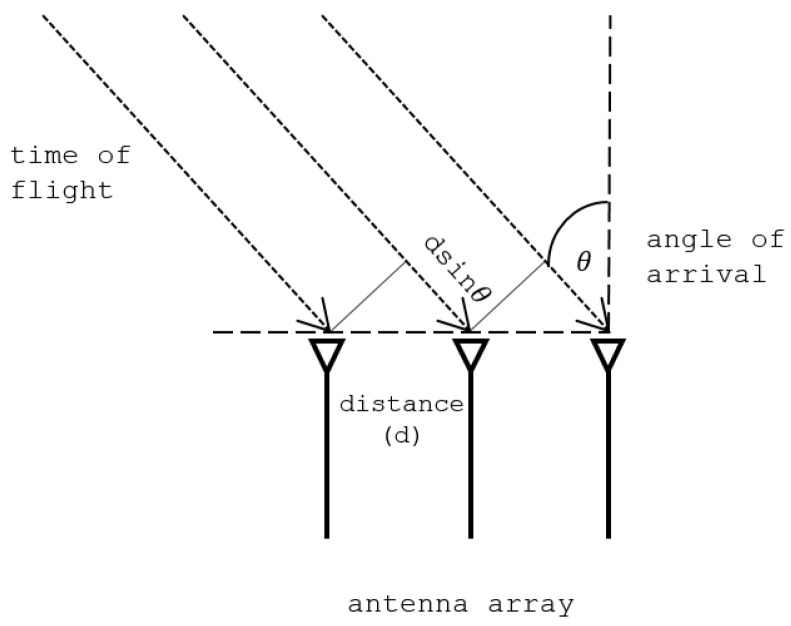
Signal impinging on a linear antenna array.

In the first instance, let us consider the signal for a single path from the transmitter to the receiver. Furthermore, let the ToF from the transmitter to the receiver be τ and the AoA (see Figure 1) be θ. In our case, we have three antennas separated by the distance *d* (typically half the wavelength λ) and each of the antennas receives *n* signals, one for each of the subcarriers whose frequencies are separated by δ. Let us denote the CSI value of the subcarrier with the lowest frequency on the first antenna by the complex number γ. It then follows that the CSI value of the *s*th subcarrier received at the *a*th antenna is $\gamma x^{a-1}y^{s-1}$, where $x=e^{-2\pi jd\lambda^{-1}\sin\theta }$ and $y=e^{-2\pi j\delta \tau }$. The CSI values associated with all three antennas and *n* subcarriers can be collected in the CSI matrix as

$$C=\left[\begin{array}{c} 1\\ x\\ x^{2} \end{array}\right]\gamma \left[\begin{array}{cccc} 1 & y & \cdots & y^{n-1} \end{array}\right].$$

When there are *p* multipath components, the corresponding CSI matrix is obtained by superposition as

(1) $$C=\left[\begin{array}{cccc} 1 & 1 & \cdots & 1\\ x_{1} & x_{2} & \cdots & x_{p}\\ x_{1}^{2} & x_{2}^{2} & \cdots & x_{p}^{2} \end{array}\right]\mathrm{diag}\left[\gamma_{1},\gamma_{2},\cdots ,\gamma_{p}\right]\left[\begin{array}{cccc} 1 & y_{1} & \cdots & y_{1}^{n-1}\\ 1 & y_{2} & \cdots & y_{2}^{n-1}\\ \vdots & \vdots & \ddots & \vdots \\ 1 & y_{p} & \cdots & y_{p}^{n-1} \end{array}\right],$$

where $x_{k}=e^{-2\pi jd\lambda^{-1}\sin\theta_{k}}$ and $y_{k}=e^{-2\pi j\delta \tau_{k}}$ and $\theta_{k}$ and $\tau_{k}$ are the AoA and ToF of the *k*th multipath component, respectively.

As the maximum rank of the CSI matrix is three, it is not suitable in its present form for the application of subspace-based methods to estimate the AoA and ToF parameters. Therefore, we use the elements of the CSI matrix to construct an enhanced matrix that allows us to exploit the following result, which is similar to Theorem 1 [11].

**Lemma** **1.** *Let $M\in C^{2q\times p}$ be an enhanced matrix that satisfies*

$$M=\left[\frac{M_{1}}{M_{2}}\right]=\left[\frac{A}{AD}\right]T^{-1},$$

*where $M_{1},M_{2},A\in C^{q\times p}$, $D=\mathrm{diag}\left(d_{1},\cdots ,d_{p}\right)\in C^{p\times p}$, q≥p, T is a unique $N_{s}\times N_{s}$ non-singular matrix, and $rank\left(A\right)=rank\left(D\right)=rank\left(T\right)=p$. Then, we have*

$$M_{1}^{†}M_{2}=TDT^{-1},$$

*where the superscript* † *denotes the Moore–Penrose pseudo-inverse.*

**Proof.**  It is easy to show that

$$\begin{array}{cc} M_{1}^{†}M_{2} & =M_{1}^{†}ADT^{-1}\\ & =M_{1}^{†}AT^{-1}TDT^{-1}\\ & =M_{1}^{†}M_{1}TDT^{-1}\\ & =TDT^{-1}. \end{array}$$

 ☐

We now construct an enhanced matrix by modifying the construction in [11] to suit our application. The motivation here is to construct a matrix that has a greater rank than the original CSI matrix in (1) and has a structure that allows us to exploit Lemma 1 to estimate the unknown AoA and ToF parameters. The enhanced matrix is

$$C^{e}=\left[\begin{array}{cc} C_{1} & C_{2}\\ C_{2} & C_{3} \end{array}\right],$$

where

(2) $$C_{i}=\left[\begin{array}{cccc} c_{i,1} & c_{i,2} & \cdots & c_{i,r}\\ c_{i,2} & c_{i,3} & \cdots & c_{i,r+1}\\ \vdots & \vdots & \ddots & \vdots \\ c_{i,m} & c_{i,m+1} & \cdots & c_{i,m+r-1} \end{array}\right],i=1,2,3.$$

$c_{i,j}$ denotes the (i,j)th entry of *C* in (1), and the free integer parameters *m* and *r* are set such that $C_{i}$ is (almost) square while m+r−1=n. This matrix can be decomposed as

$$C^{e}=E_{L}\Gamma E_{R}^{T},$$

where

$$E_{L}=\left[\frac{Y_{m}}{Y_{m}D_{x}}\right],\;E_{R}=\left[\frac{Y_{r}}{Y_{r}D_{x}}\right],$$

(3) $$Y_{m}=\left[\begin{array}{cccc} 1 & 1 & \cdots & 1\\ y_{1} & y_{2} & \cdots & y_{p}\\ \vdots & \vdots & \ddots & \vdots \\ y_{1}^{m-1} & y_{2}^{m-1} & \cdots & y_{p}^{m-1} \end{array}\right],\;Y_{r}=\left[\begin{array}{cccc} 1 & 1 & \cdots & 1\\ y_{1} & y_{2} & \cdots & y_{p}\\ \vdots & \vdots & \ddots & \vdots \\ y_{1}^{r-1} & y_{2}^{r-1} & \cdots & y_{p}^{r-1} \end{array}\right],$$

$$\Gamma =\mathrm{diag}\left[\gamma_{1},\gamma_{2},\cdots ,\gamma_{p}\right],$$

and

$$D_{x}=\mathrm{diag}\left[x_{1},\cdots ,x_{p}\right].$$

We perform a singular value decomposition of $C^{e}$ to obtain

$$C^{e}=U\Sigma V^{H}$$

and partition the matrix of the left singular vectors as $U=\left[U^{s}|U^{\nu }\right],$ where the columns of $U^{s}$ correspond to the *p* largest left singular values. In the absence of any noise or error, we have

$$\mathrm{range}\left(U^{s}\right)=\mathrm{range}\left(E_{L}\right).$$

Therefore, there exists a non-singular complex p×p matrix, say *T*, such that

$$U^{s}=E_{L}T^{-1}.$$

If we partition $U^{s}$ as

$$U^{s}=\left[\frac{U_{1}^{s}}{U_{2}^{s}}\right]=\left[\frac{Y_{m}}{Y_{m}D_{x}}\right]T^{-1}$$

and have $\mathrm{rank}\left(U_{1}^{s}\right)=p$, it follows from Lemma 1 that

$${\left(U_{1}^{s}\right)}^{†}U_{2}^{s}=TD_{x}T^{-1}.$$

Hence, the eigenvalues of ${\left(U_{1}^{s}\right)}^{†}U_{2}^{s}$ are $x_{1},\cdots ,x_{p}$.

The singular value decomposition for $C^{e}$ can be used to calculate $y_{1},\cdots ,y_{p}$ as well. This is due to the following property:

$$P\left(\left[\begin{array}{c} 1\\ x \end{array}\right]⊗\left[\begin{array}{c} 1\\ y\\ \vdots \\ y^{m-1} \end{array}\right]\right)=\left[\begin{array}{c} 1\\ y\\ \vdots \\ y^{m-1} \end{array}\right]⊗\left[\begin{array}{c} 1\\ x \end{array}\right],$$

where *P* is a permutation matrix with the *i*th entry of its corresponding permutation vector being equal to $\left(i+1\right)/2$ or i/2+m, for odd or even entries, respectively.

We define the matrices ${\hat{Y}}_{L1}$ and ${\hat{Y}}_{L2}$ by removing the last two and first two rows of $PE_{L}$, respectively, as

$${\hat{Y}}_{L2}={\hat{Y}}_{L1}D_{y}$$

with

$$D_{y}=\mathrm{diag}\left[y_{1},\cdots y_{p}\right].$$

We also define the matrices ${\hat{U}}_{1}^{s}$ and ${\hat{U}}_{2}^{s}$ by removing the last two and first two rows of $PU^{s}$, respectively. Then, similar to the previous step, we have

$${\hat{U}}^{s}=\left[\frac{{\hat{U}}_{1}^{s}}{{\hat{U}}_{2}^{s}}\right]=\left[\frac{{\hat{Y}}_{L1}}{{\hat{Y}}_{L1}D_{y}}\right]T^{-1},$$

and, subsequently, if $\mathrm{rank}\left({\hat{U}}_{1}^{s}\right)=p$,

$${\left({\hat{U}}_{1}^{s}\right)}^{†}{\hat{U}}_{2}^{s}=TD_{y}T^{-1},$$

which means that the eigenvalues of ${\left({\hat{U}}_{1}^{s}\right)}^{†}{\hat{U}}_{2}^{s}$ are $y_{1},\cdots ,y_{p}$. Note that the eigenvectors of ${\left(U_{1}^{s}\right)}^{†}U_{2}^{s}$ and ${\left({\hat{U}}_{1}^{s}\right)}^{†}{\hat{U}}_{2}^{s}$ are the same. This observation is used in [10] to match the estimated values of $x_{1},\cdots ,x_{p}$ and $y_{1},\cdots ,y_{p}$.

### 3.2. Pairing the AoA and ToF Estimates

Denote the eigenvectors of ${\left(U_{1}^{s}\right)}^{†}U_{2}^{s}$ and ${\left({\hat{U}}_{1}^{s}\right)}^{†}{\hat{U}}_{2}^{s}$ by $w_{k}$, k=1,2,⋯,p. If we estimate the values of $y_{k}$ and $w_{k}$, k=1,2,⋯,p, from ${\left({\hat{U}}_{1}^{s}\right)}^{†}{\hat{U}}_{2}^{s}$, we will have

$$x_{k}U_{1}^{s}w_{k}=U_{2}^{s}w_{k}.$$

Then, by multiplying both sides by $w_{k}^{H}{U_{1}^{s}}^{H}$ from the left, we get

$$x_{k}w_{k}^{H}{U_{1}^{s}}^{H}U_{1}^{s}w_{k}=w_{k}^{H}{U_{1}^{s}}^{H}U_{2}^{s}w_{k}$$

and, consequently,

(4) $$x_{k}=\frac{w_{k}^{H}{U_{1}^{s}}^{H}U_{2}^{s}w_{k}}{w_{k}^{H}{U_{1}^{s}}^{H}U_{1}^{s}w_{k}}.$$

This way, we obtain the values of $x_{k}$ correctly paired with the values of $y_{k}$ for each multipath component.

### 3.3. Some Notable Challenges

The use of CSI data for the estimation of AoA and ToF values of multipath components has some limitations. The typical hostility of indoor propagation environments coupled with limited bandwidth of WiFi channels makes the estimation process challenging. Mainstream WiFi systems specified by the IEEE 802.11 a/g/n standards have bandwidths up to 40 MHz per channel. Using an Intel 5300 WiFi network interface card, it is possible to extract CSI values of 30 subcarriers with a narrow subcarrier spacing. It is known that subspace-based parameter estimation techniques perform poorly when applied to closely-spaced sinusoids [35].

The limited bandwidth and narrow subcarrier spacing make the matrix $Y_{m}$ in (3) poorly conditioned. Therefore, it is hard to detect the AoA and ToF of all the multipath components in a complex and noisy environment. The chances of detecting the AoA and ToF of the multipath components depend on their contribution in the CSI matrix, which is influenced by the amplitude of the multipath components. The signal traveling in the line of sight (LoS) path has a higher amplitude that leads to a higher contribution in the CSI matrix compared to non-LoS signals. Multipath components with higher amplitude than the ambient noise give a higher condition number in the CSI matrix. However, in practice, there is only one LoS and others are reflected, refracted, scattered, or diffracted multipath components. As a result, the CSI matrix is generally poorly conditioned.

The signal traveling via the shortest path from the transmitter to the receiver, which is the LoS path, represents the AoA that can be utilized for localization. This signal component is typically unique, if it exists. However, there may be multiple multipath components that arrive at the receiver with the same AoA. The direct application of the MMP algorithm in such cases results in large errors when estimating the ToF of the multipath components with the same AoA. The reason is that the MMP algorithm is designed for rectangular antenna arrays with the number of elements being higher than the number of impinging signals. In our re-formulation using the CSI matrix, $U_{1}^{s}$ is rank-deficient. However, ${\hat{U}}_{1}^{s}$ is not rank-deficient, which allows us to estimate the AoA values accurately. We solve the problem by simply estimating the AoA–ToF pairs in the opposite order, i.e., multiplying the signal subspace by the permutation matrix to estimate the ToF values first. This way the outcome is not affected by events when some AoAs are equal.

## 4. Multi-Packet CSI Aggregation

To better cope with the uncertainties of indoor environments, the CSI values associated with multiple OFDM packet transmissions can be measured and used for estimating the AoA and ToF of multipath components. In [6], the CSI values from 170 packets are separately processed at each access point to estimate the AoA of the path with the shortest ToF in a noisy environment. The WiFi card used there can communicate 2000 packets every second. Utilizing CSI from a higher number of packets generally delivers more accurate estimation of AoA and ToF.

In this section, we propose a CSI aggregation method that uses the CSI values associated with multiple packet transmissions to construct an aggregate CSI data matrix that has improved SNR and leads to a more accurate estimation of the ToF and AoA of the multipath components. In addition, it can speed up the process as it entails carrying out the estimation only once for multiple packets. We describe the method in the following.

Suppose we have the CSI data for *L* packets and denote the *l*th one by $d_{l}\in C^{3n\times 1}$ in a vectorized format and express it as

$$\begin{array}{cc} d_{l} & =\sum_{k=1}^{p}a\left(\theta_{k},\tau_{k}\right)\omega_{lk}+\nu_{l}\\ & =A\left(\psi \right)\omega_{l}+\nu_{l},\;l=1,\cdots ,L, \end{array}$$

where

$$\omega_{l}={\left[\omega_{l1},\cdots ,\omega_{lp}\right]}^{T},$$

$$A\left(\psi \right)=\left[a\left(\theta_{1},\tau_{1}\right),a\left(\theta_{2},\tau_{2}\right),\cdots ,a\left(\theta_{p},\tau_{p}\right)\right],$$

$$\psi =\left[\theta_{1},\tau_{1},\theta_{2},\tau_{2},\cdots ,\theta_{p},\tau_{p}\right],$$

and $\nu_{l}$ is the noise (perturbation) vector.

The vectors of complex amplitudes $\omega_{l}$, l=1,⋯,L are different for each packet due to the presence of sampling time and frequency offsets in commercial WiFi systems [6]. Therefore, we can write $\omega_{l}=\beta_{l}\omega$ using some arbitrary values of $\beta_{l}\in C$, l=1,⋯,L, and a packet-independent vector $\omega \in C^{p\times 1}$ to obtain

$$d_{l}=\beta_{l}A\left(\psi \right)\omega +\nu_{l},\;l=1,\cdots ,L.$$

A possible way of using $d_{l}$, l=1,⋯,L to alleviate the adverse effects of noise is by taking their average. However, this may not increase the SNR since $\beta_{l}$, l=1,⋯,L, also bears uncertainty. An alternate approach that we propose is to calculate the singular value decomposition

$$D=\left[d_{1},d_{2},\cdots ,d_{L}\right]=U\Sigma V^{H}$$

and take the left singular vector corresponding to the largest eigenvalue as the aggregate CSI data associated with the considered *L* packets. We may use this aggregate CSI data in place of the CSI data of the multiple packets to perform the AoA and ToF estimation only once yet with improved performance. We will demonstrate the advantages of this approach in the next section.

## 5. Simulation Results

In this section, we evaluate the proposed MMP algorithm in terms of estimation and computational performance. We first compare the MMP algorithm with the 2D MUSIC algorithm in noisy and noise-free scenarios. We then evaluate the performance of MMP in the event of some multipath components having the same AoA and evaluate the effects of changing the oder of AoA and ToF estimation. The simulations are done using MATLAB. For simulating multipath propagation, we use the Wireless Insite software [36].

The parameters that we use in our simulations are similar to those used for the 2D MUSIC in [6]. The 2D MUSIC requires a pre-defined set of AoA and ToF values for which the 2D MUSIC spectrum is calculated. Moreover, it requires a peak search to find the local maxima of the spectrum. The resolution of the considered AoA–ToF grid and the employed peak-search algorithm are the key factors determining the overall computational complexity of the 2D MUSIC algorithm. We use an efficient peak-search technique that provides an error-free detection of the peaks. To this end, we convert the 2D peak search process into a combination of 1D searches by first finding the highest values across the ToF axis, then across the AoA axis.

Table 1 shows the values of the parameters used in our simulations. We use the WiFi channel number 126 that has a bandwidth of 40 MHz and the center frequency of 5.63 GHz. Using the Intel 5300 NIC, we have access to the CSI values of 30 subcarriers in this channel.

Similar to [6], we take 101 points for AoA from −90 to 90 degrees, which gives a resolution of 1.78 degrees. For ToF, the resolution is 0.5 nanosecond (ns), which corresponds to around 14.83 cm in terms of the traveled distance.

**Figure 2:**
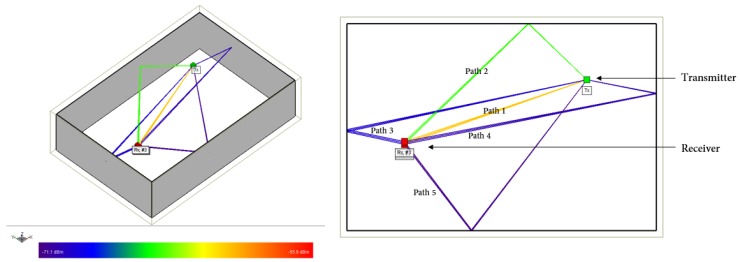
Multipath propagation simulated by the Wireless Insite software.

Figure 2 shows an event of multipath propagation with one LoS and four non-LoS components simulated by the Wireless Insite software. Signals traveling in different directions are reflected from the wall surfaces and decay in intensity. Compared to the LoS component, the non-LoS components travel longer distances and are attenuated more severely. Table 2 shows the received signal strength indicator (RSSI), AoA, and ToF of all the multipath components shown in Figure 2. The components are listed by their RSSI values in the descending order. Path 1 is the LoS path and has the highest RSSI value.

In the 2D MUSIC algorithm, the AoA and ToF of the multipath components are estimated by detecting the peaks of the MUSIC spectrum. A multipath component with a small amplitude usually produces a small and rather smooth peak in the MUSIC spectrum. In a noisy environment, it is hard to detect a clear peak if the amplitude of the multipath component is low. In our experiments with simulated indoor propagation environments, the number of distinguishable peaks on the MUSIC spectrum is often not greater than three. Therefore, in our simulations and in the presence of noise, we consider the number of significant and detectable multipath components to be three. Clearly, this number depends on the available bandwidth, subcarrier spacing, SNR, complexity of the propagation environment, etc. As discussed before, a higher bandwidth gives a CSI matrix with a better conditioning where the ratios between the singular values are small and the multipath components with significant contribution can be identified more accurately.

**Figure 3:**
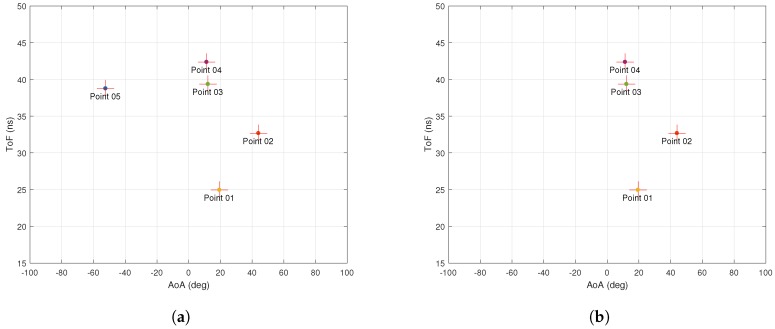
Simulation with no noise. (**a**) performance of MMP with no noise. Simulation time is 0.044 s; (**b**) performance of 2D MUSIC with no noise. Simulation time is 8.66 s.

**Figure 4:**
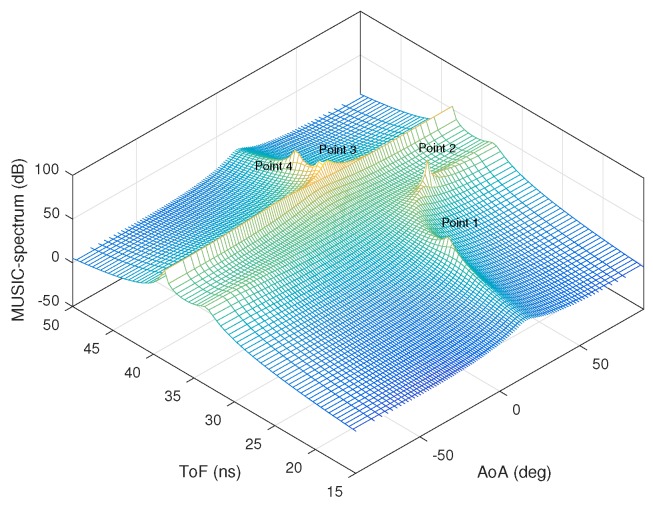
2D Music spectrum with no noise.

Figure 3a,b shows the AoA and ToF values estimated by the MMP and 2D MUSIC algorithms in the absence of ambient noise. The dots represent the estimated AoA–ToF values and the crosses are at the true AoA–ToF coordinates. Since there is no noise, the MMP algorithm is able to detect all five multipath components. However, the 2D MUSIC algorithm detects only four of them since the fifth component does not create any sufficiently pronounced peak on the MUSIC spectrum shown in Figure 4.

### 5.1. Without Ambient Noise

Apart from that, both algorithms demonstrate similar estimation performance. The main difference is in their computational complexities. The CPU time and the number of floating point operations (FLOPs) required by a single run of each algorithm ignoring the complexity of peak search in 2D MUSIC is given in Table 3. The FLOP counts are due to a Matlab tool from [37]. It is evident from Table 3 that MMP is significantly (around 200 times) faster than 2D MUSIC in this experiment.

### 5.2. With Ambient Noise

**Figure 5:**
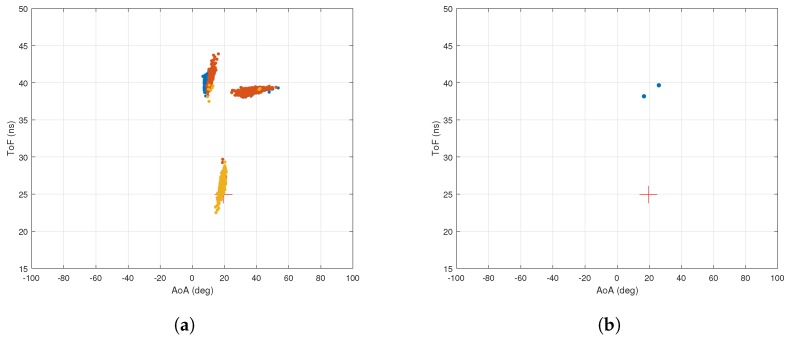
Simulation with ambient noise. (**a**) performance of MMP with SNR=35 dB for 1000 runs. The RMSEs for AoA and ToF are 2.34 deg and 6.24 ns and the bias for AoA ToF are −1.53 deg and −0.49 ns. Simulation time is 0.79 s; (**b**) performance of 2D MUSIC with SNR=35 dB for 1000 runs. The RMSEs for AoA and ToF are 2.60 deg and 13.69 ns and the biases for AoA and ToF are −1.97 deg and 16.95 ns. Simulation time is 152.21 s.

**Figure 6:**
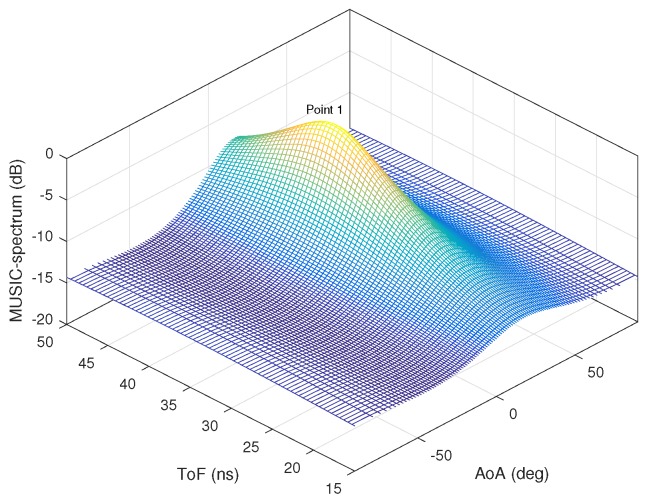
2D Music spectrum with SNR=35 dB.

Next, we consider the CSI values to be contaminated with additive Gaussian noise with zero mean and variance set according to the desired signal-to-noise ratio (SNR). In both MMP and 2D MUSIC algorithms, we assume three dominant multipath components. This means we consider the rank of $U_{1}^{s}$ to be three. Figure 5a,b shows the AoA and ToF values estimated by the MMP and 2D MUSIC algorithms for 1000 independent runs when SNR is 35 dB. In these figures, the cross marks the true AoA–ToF of the LoS component. Figure 6 shows the associated MUSIC spectrum.

The results for MMP show a bias of −1.13 deg in the estimated AoA and a bias of 2.49 ns in the estimated ToF for the LoS path that is the one with the smallest ToF. The 2D MUSIC algorithm extracts only two peaks from its spectrum in all 1000 runs. In Figure 5b, the bias for AoA is almost the same as in Figure 5a. However, 2D MUSIC has a higher root mean square error (RMSE) for AoA compared to MMP. The estimated AoA–ToF values by 2D MUSIC do not show any variance.

**Figure 7:**
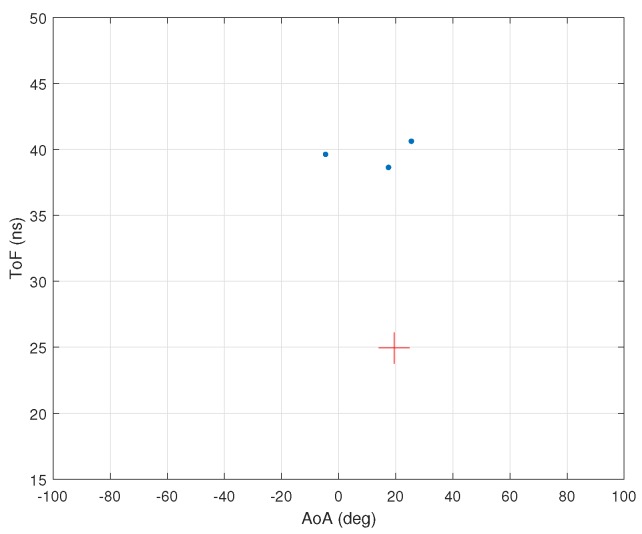
Performance of 2D MUSIC with SNR=35 dB and a higher resolution gird for 1000 runs. The RMSEs for AoA and ToF are 1.97 deg and 13.69 ns. Simulation time is 201.45 s.

The limited resolution in the grid over which the MUSIC spectrum is calculated makes it difficult for 2D MUSIC to find the highest peak at its exact location. Figure 7 shows the results when we use a higher resolution (201 values) for both AoA and ToF to construct the MUSIC spectrum. The finer resolution helps find more peaks in the MUSIC spectrum at the cost of increased estimation time of 207.45 s. Figure 7 shows three peaks and a lower RMSE in the estimated AoA values. However, it appears that the accuracy of ToF estimation is not promising when there is ambient noise.

**Figure 8:**
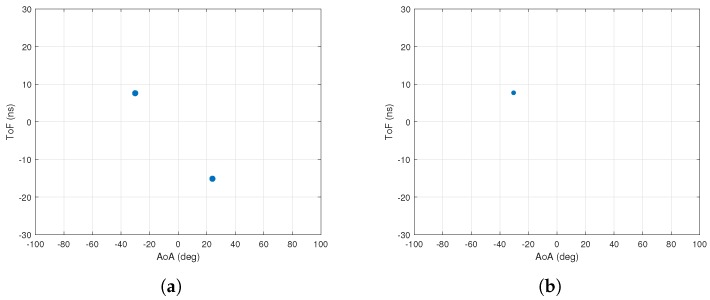
Performance using CSI from experiment. (**a**) performance of MMP with real CSI data. Algorithm runtime is 0.013 s; (**b**) performance of 2D MUSIC with real CSI data. Algorithm runtime is 2.24 s.

Figure 8a,b shows the performance of MMP and 2D MUSIC using real CSI data collected from an Atheros AR9590 network interface card. The modified kernel given in [38] allows for collecting CSI data of 56 sub-carriers corresponding to each receiver antenna for each packet. In the experiment, we placed the transmitter antenna at an angel of approximately −42 degrees with respect to the receiver. The required post-processing was performed as discussed in [6]. We observe in Figure 8a,b that MMP correctly estimates the AoA and ToF of the direct path as well as those of a dominant indirect path while 2D MUSIC estimates the AoA and ToF of the direct path only as it discovers only one peak in its spectrum, which corresponds to the direct path.

### 5.3. Lower Grid Resolution

**Figure 9:**
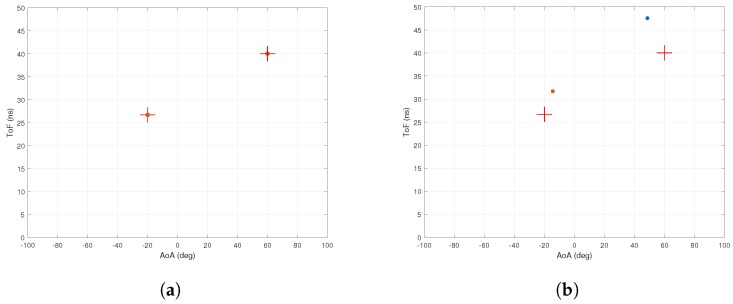
Simulation with no noise. (**a**) performance of MMP with SNR=35 dB for 1000 runs. The RMSEs for AoA and ToF are 0.057 deg and 0.023 ns. Simulation time is 0.83 s; (**b**) performance of 2D MUSIC with SNR=35 dB and a lower resolution grid for 1000 runs. The RMSEs for AoA and ToF are 8.4661 deg and 6.271 ns. Simulation time is 2.3 s.

**Figure 10:**
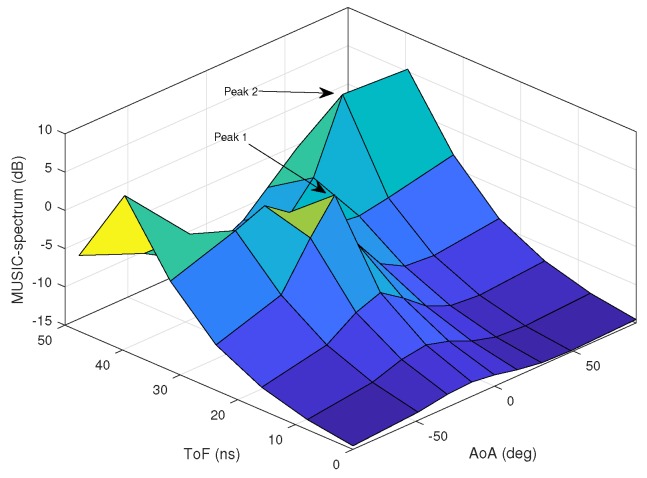
2D MUSIC spectrum with SNR=35 dB and a lower grid resolution.

Setting a lower resolution for the spectrum grid of 2D MUSIC alleviates its computational complexity. However, it also significantly compromises its estimation performance. We run another set of simulations with only two multipath signals. Figure 9a,b shows the AoA and ToF values estimated by the MMP and 2D MUSIC for 1000 independent runs when SNR is 35 dB and the grid resolution for the 2D MUSIC spectrum is reduced to nine points for AoA and seven points for ToF as illustrated in Figure 10. As seen in Figure 9a,b, a low resolution in the MUSIC spectrum can drastically reduce the accuracy of 2D MUSIC. We use the above grid resolution to make the runtime of 2D MUSIC close to that of MMP.

### 5.4. Larger Channel Bandwidth

**Figure 11:**
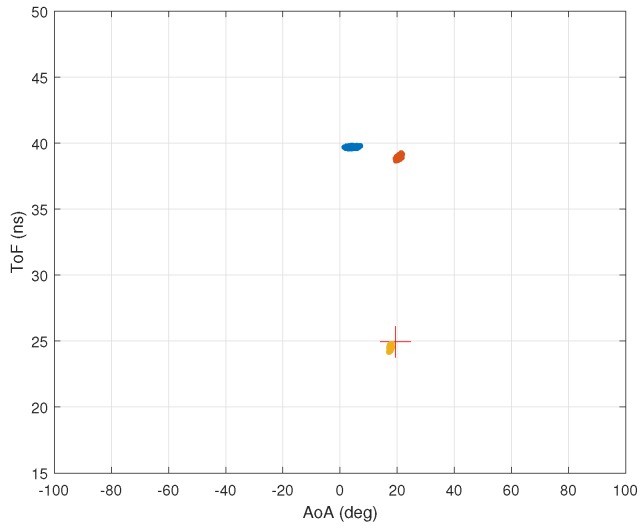
Performance of MMP when bandwidth is 80 MHz for 1000 runs. The RMSEs for AoA and ToF are 1.80 deg and 0.44 ns. Simulation time is 0.80 s.

The limited bandwidth of a typical WiFi channel makes the CSI data matrix poorly conditioned. Therefore, the use of a higher bandwidth can increase the chances of detecting a higher number of multipath components and improve the accuracy of estimating the AoA and ToF of the detected multipath components. Figure 11 shows the estimated AoA–ToF values for the same scenario as in Figure 5a but using a bandwidth of 80 MHz instead of 40 MHz. The higher bandwidth obviously improves the performance as the RMSE of AoA reduces from 2.34 degrees to 1.80 degrees.

### 5.5. Order of Estimation

A simple implementation of the MMP algorithm first estimates the AoA values, then the ToF values.

This order of estimation is prone to gross errors when some multipath components have the same AoA. We can resolve this issue by changing the order of the eigenvalue decompositions. That means instead of estimating the AoA first, we calculate the ToF and then estimate the values of AoA using (4). This way, MMP performs better with multiple multipath components having the same AoA.

**Figure 12:**
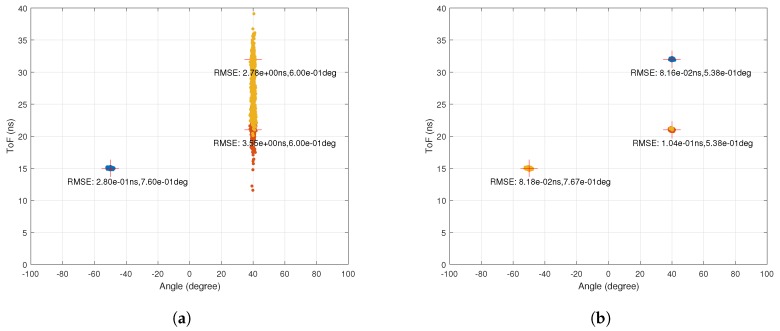
Ordering performance. (**a**) performance of MMP estimating AoA first for 1000 runs. The Average RMSEs for ToF and AoA are 2.20 ns and 0.65 deg; (**b**) performance of MMP estimating ToF first for 1000 runs. The RMSE for ToF and AoA are 0.089 ns and 0.61 deg.

Figure 12a shows the AoA–ToF values estimated by the MMP algorithm when two multipath components have the same AoA. In this experiment, we have three multipath components with the same intensity.

The AoA of two multipath components are the same. As seen in Figure 12a, the AoA values are estimated with good accuracy but not the ToF values. Figure 12b shows the AoA–ToF values estimated by the MMP algorithm using the reverse order. The figure clearly illustrates the advantage of the reversed order.

### 5.6. Multi-Packet CSI Aggregation

**Figure 13:**
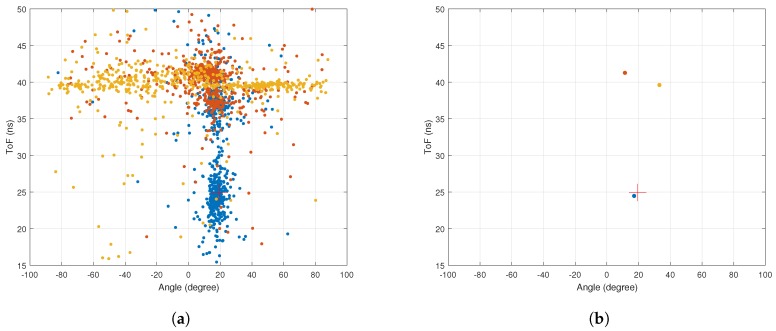
Multi-packet CSI aggregation performance. (**a**) performance of MMP with SNR=20 dB for 1000 runs. The RMSEs for AoA and ToF are 12.39 deg and 22.92 ns. Simulation time is 0.76 s; (**b**) performance of MMP using the multi-packet CSI aggregation method when SNR=20 dB for one run using CSI of 1000 packets. The RMSEs for AoA and ToF are 2.29 deg and 0.46 ns. Simulation time (including CSI aggregation) is 0.14 s.

Figure 13a,b shows the AoA–ToF values estimated by the MMP algorithm with and without using the proposed multi-packet CSI aggregation method when CSI for 1000 packets is available and the SNR is 20 dB in the scenario simulated in Figure 5a. It is clear that aggregating the CSI of 1000 packets significantly improves the estimation performance.

## 6. Conclusions

A recent indoor localization method, called SPOTFI, has applied the subspace-based 2D MUSIC algorithm for estimating the AoA and ToF of multipath components. We showed that a modified matrix pencil (MMP) algorithm, which has originally been developed for estimating two-dimensional angles using a rectangular antenna array and does not require any grid search, is an effective alternative to the 2D MUSIC algorithm. Our numerical experiments demonstrated that using MMP in place of 2D MUSIC speeds up the joint AoA–ToF estimation of multipath components by around 200 times while delivering similar estimation accuracy in typical indoor scenarios. Moreover, we showed that changing the order of AoA and ToF estimation in the MMP algorithm improves the performance when there are two or more multipath components with the same AoA. In addition, we proposed a method for aggregating the CSI from multiple packets to enhance the quality of the CSI data. Instead of estimating the AoA and ToF values multiple times using the CSI from multiple packets, using the proposed multi-packet CSI aggregation method, all the available CSI data can be combined to carry out the estimation only once. We showed via simulations that this way both estimation accuracy and computational efficiency are improved. The theoretical performance analysis of the MMP algorithm for the problem considered in this paper can be an interesting topic for future work. Particularly, characterizing the performance versus various governing factors such as the SNR and channel bandwidth can provide valuable insights into the strengths and limitations of the algorithm as well as guidelines on its effective utilization in practice.

## Figures and Tables

**Table 1:** Simulated parameters.

Parameter	Value
radio frequency	5.63 GHz
bandwidth	40 MHz
number of subcarriers	30
AoA range	−90 to + 90 degree
AoA steps (for 2D MUSIC)	101
ToF range	0 to 50 ns
ToF steps (for 2D MUSIC)	101

**Table 2:** Parameter values of the multipath components.

Component	RSSI (dBm)	AoA (deg)	ToF ($10^{-8}$)
path 1	−60.603	19.4553	2.49486
path 2	−64.391	44.0316	3.267340
path 3	−69.270	167.794	3.93585
path 4	−69.976	11.3285	4.23677
path 5	−70.797	−52.3761	3.87655

**Table 3:** The required number of floating point operations in a single run.

	2D MUSIC	MMP
time (ms)	8.66	0.04
FLOPs ($\times 10^{6}$)	381	2.17
